# Robust, universal, and persistent bud secretion adhesion in horse-chestnut trees

**DOI:** 10.1038/s41598-020-74029-5

**Published:** 2020-11-04

**Authors:** Dagmar Voigt, Jaekang Kim, Anne Jantschke, Michael Varenberg

**Affiliations:** 1 Institute for Botany, Faculty of Biology, Technische Universität Dresden, 01062 Dresden, Germany; 2 George W. Woodruff School of Mechanical Engineering, Georgia Institute of Technology, Atlanta, GA 30332 USA; 3 Bioanalytical Chemistry, Technische Universität Dresden, 01062 Dresden, Germany

**Keywords:** Biochemistry, Biophysics, Plant sciences, Materials science, Electron microscopy

## Abstract

Buds of horse-chestnut trees are covered with a viscous fluid, which remains sticky after long-term exposure to heat, frost, radiation, precipitation, deposition of aerosols and particles, attacks by microbes and arthropods. The present study demonstrates that the secretion does not dry out under arid conditions, not melt at 50 °C, and not change significantly under UV radiation or frost at a microscopic level. It is slightly swellable under wet conditions; and, it universally wets and adheres to substrates having different polarities. Measured pull-off forces do not differ between hydrophilic and lipophilic surfaces, ranging between 58 and 186 mN, and resulting in an adhesive strength up to 204 kPa. The mechanical and chemical properties of secretion resemble those of pressure-sensitive adhesives. The Raman, infrared, and nuclear magnetic resonance spectra show the clear presence of saturated aliphatic hydrocarbons, esters, free carboxylic acids, as well as minor amounts of amides and aromatic compounds. We suggest a multi-component material (aliphatic hydrocarbon resin), including alkanes, fatty acids, amides, and tackifying terpenoids embedded in a fluid matrix (fatty acids) comprising nonpolar and polar portions serving the universal and robust adhesive properties. These characteristics matter for ecological-evolutionary aspects and can inspire innovative designs of multifunctional, biomimetic pressure-sensitive adhesives and varnishes.

## Introduction

The glistening buds of horse-chestnut trees are covered with a viscous fluid, which is present from the early bud development until its unfolding after about 10–11 months, surviving summer, autumn, and winter conditions^1,2^. This fluid is secreted by the so-called colleters (spherical multicellular glandular trichomes). It spreads over the inner and outer surfaces of six to eight decussate pairs of bud scales, filling the bud scale interspaces and creating a multilayered impregnated structure^1,3,4^. This material protects the developing meristems of the shoot apex and floral buds against environmental stresses, such as high evapotranspiration and dehydration, e.g.,^5–10^. Highly reflective plant surfaces may exhibit a marked reduction in water loss through lowering the temperature of a leaf by 10–15 °C and thus, decreasing the transpiration rate^11–13^. Plant surface mucilages may play a role in water retention due to their ability to absorb water^14–16^. Moreover, superficial plant exudates can repel herbivores and pathogens^13,17–20^, or protect against irradiation^21^.

The viscous fluid released by the horse-chestnut bud colleters is assumably a mixture of resin or oil and gum particles soluble in acetone, e.g.,^1–3,22^. Acetone or hexane extractions of entire buds and subsequent chromatography analyses (GC–MS) reveal a heterogeneous, chemically diverse material composed of a nonpolar portion (crystallizing flavonols, small amounts of triterpenes) and a polar syrup-like portion, which is the main mass of the extract. Larger fractions of methylated flavonoid aglycones, such as kaempferoltri- and dimethylether, quercetindi- and trimetylether, myricetin trimethylether, rhamnazin, rhamnocitrin, kaempferide, and isorhamnetin have also been detected^23–28^. Besides antimicrobial properties, flavonoids can absorb the most energetic solar wavelengths (i.e., UV-B, UV-A), decreasing oxidative stress^29^.

**Figure 1:**
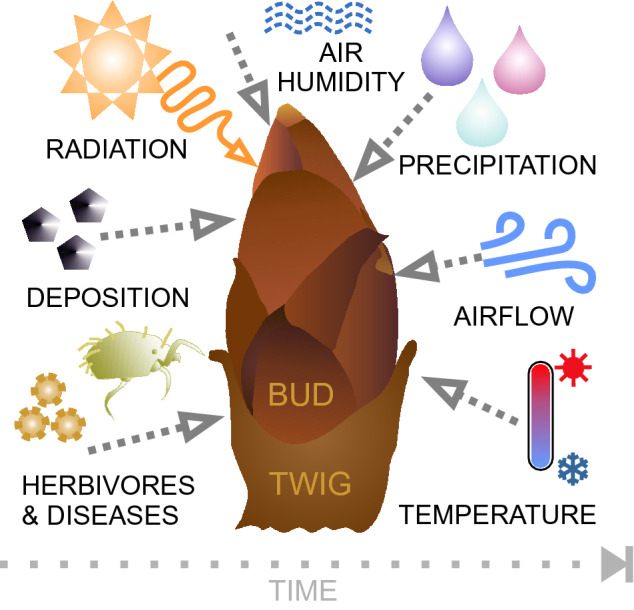
Buds of horse-chestnut trees are exposed to a variety of external abiotic and biotic impacts, such as radiation (UV, IR, etc.), humidity and temperature fluctuations, airflow, rain, snow, hail, particle and aerosol deposition, and phytosanitary risks (microbes, arthropods).

This secretion also exhibits a remarkable stickiness; once touched, it is difficult to remove from skin, clothes, and other materials. However, despite biological adhesion attract much research interest lately, plant adhesives received little attention so far, and adhesive properties of horse-chestnut tree secretions remain largely unknown. To this end, knowing about the exposure of horse-chestnut buds to a variety of impacts over time (Fig. 1), we studied the microscopic structure, chemical composition, and, in particular, the adhesive properties of the bud secretion in the context of environmental changes.

## Results

### Appearance

**Figure 2:**
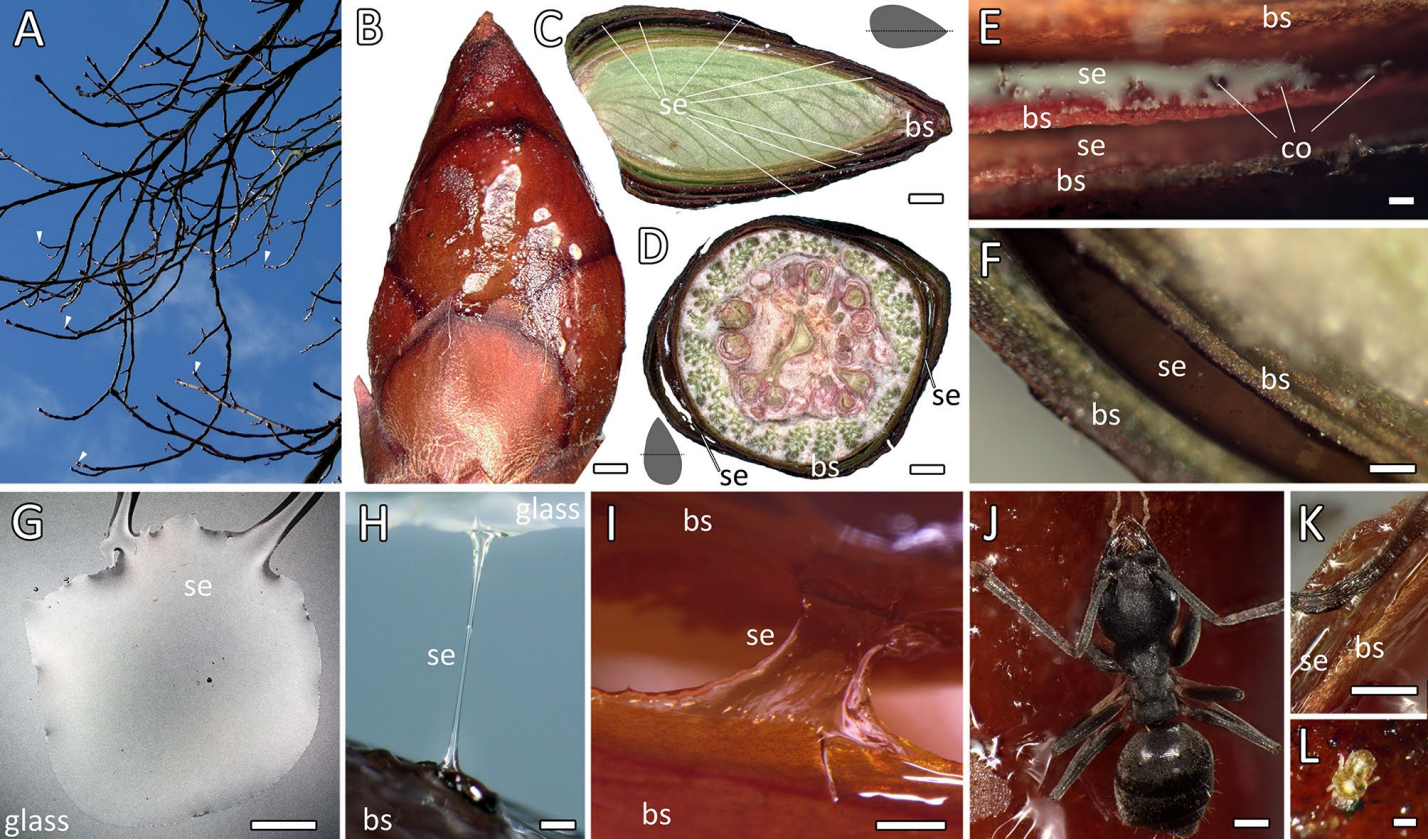
(**A**) A photograph showing horse-chestnut tree branches and twigs with distally situated, glistening buds (arrowheads). (**B**–**D**) Digital microscopic images of the lateral view (**B**), as well as the longitudinal (**C**) and transverse (**D**) cross-sections of the horse-chestnut tree bud. The shiny layer of secretion on the bud surface and between bud scales is clearly visible. (**E**,**F**) Details of the secretion embedding the bud scales. The secretion-releasing colleters are indicated in E. (**G**) Top view of a bud secretion droplet on the normal glass. (**H**,**I**) The secretion can be pulled into thin (**H**) or lamellate filaments (**I**); (**G**–**I**) stereomicroscopic images. (**J**–**L**) Digital microscopic images of a white-footed ant adhering to the bud secretion (**J**,**K**) and a mite immobilized by the sticky bud secretion (**L**). The distal appendages of arthropods are sunken in and covered with the secretion layer. *bs* bud scale, *co* colleter, *se* secretion. Scale bars: (**B**–**D**) 1000 µm, (**E**) 50 µm, (**F**–**H**,**J**,**K**) 200 µm, (**I**) 500 µm, (**L**) 100 µm.

The translucent secretion covered horse-chestnut tree buds (Fig. 2A), forming a relatively homogenous 30-to-50-µm thick superficial layer, and it was also internally present between the bud scales, filling 10-to-150-µm wide spaces between them (Fig. 2B–F). On the glass, it established flat hemispherical droplets (Fig. 2G). The secretion could be pulled into 5-cm long filaments (Fig. 2H,I). In the field, the bud surface mostly looked shiny and non-contaminated, and buds were vital and not damaged (Fig. 2A,B). At times, particles, fibers, or smaller arthropods stuck to the superficial secretion layer (Fig. 2J–L). During rain events, water droplets, if not coalescent, adhered to the bud surface and evaporated later, but never (or seldom) rolled-off.

**Figure 3:**
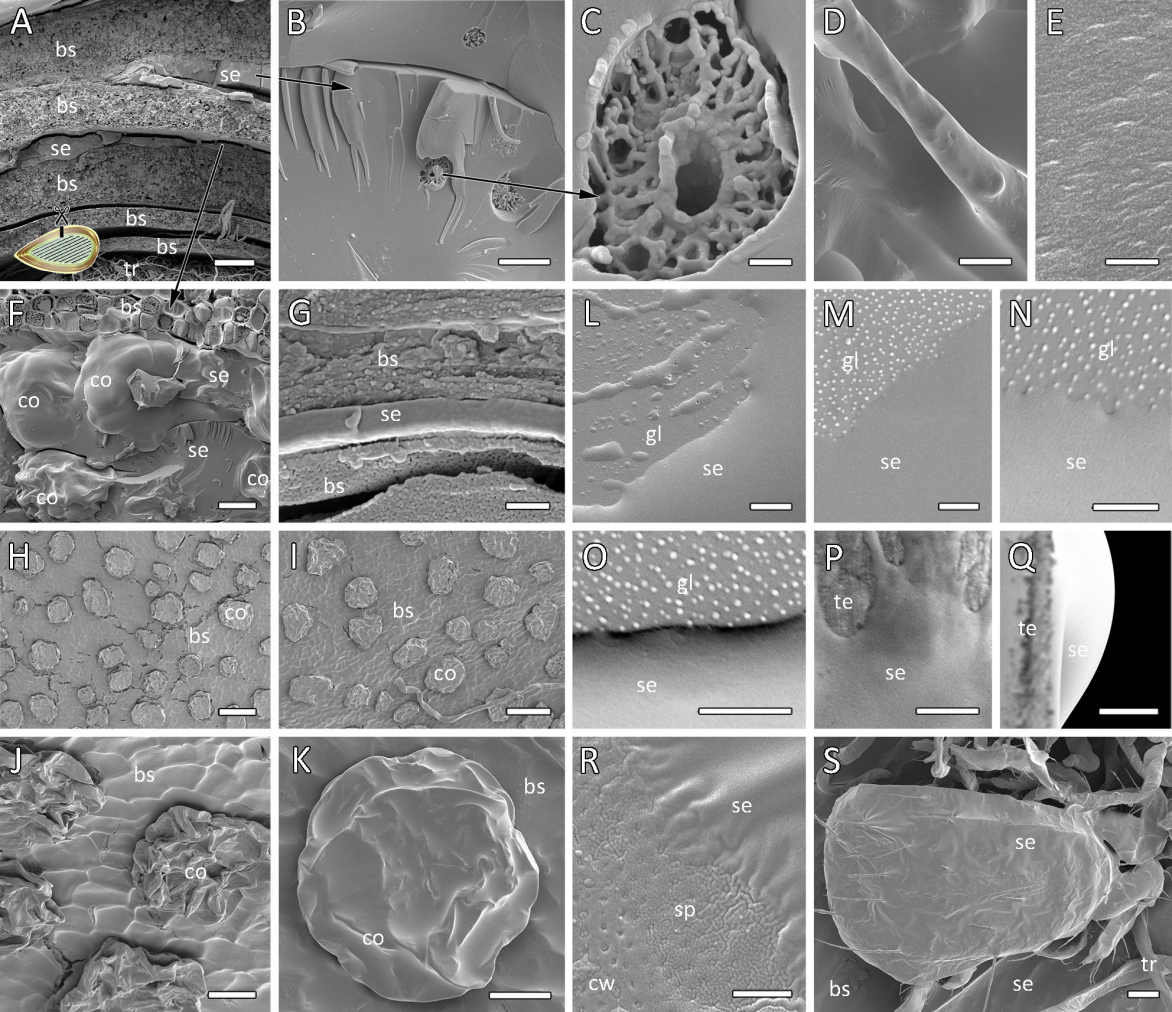
Cryo-SEM images of the horse-chestnut bud scales and secretion. (**A**) Cross-section (freeze-fracture) of bud scales and embedded secretion. The inset indicates the fracture position on the bud. See the close-up views in (**F**) and (**G**). (**B**–**E**) Details of the bud secretion cross-section after freeze-fracture (**B**,**C**), pulled into a filament indicating a fibrous component (**D**), and the non-structured surface (top view in **E**). Note the presence of micelle-shaped structures included in the amorphous layer of secretion (**B**,**C**). (**F**,**G**) Freeze fracture of bud scales and secretion covering the secretion-releasing colleters. (**H**–**K**) Solvent treatments: the bud scale surface covered with numerous collapsed colleters after washing with acetone and total removal of secretion (**H**,**I**), partially collapsed colleters after washing with ethanol leaving residues of secretion (**J**,**K**). (**L**–**O**) The interface of bud secretion droplets and normal glass, after keeping the secretion at 22.7 °C, 46.6% RH (**H**,**I**), at 22.6 °C, 0.3% RH (**J**), and at 22.4 °C, 99.3% RH (**M**). (**P**,**Q**) The secretion droplet in contact with Teflon tape, top (**P**) and side view (**Q**). (**R**) The interface between the bud secretion and a cockroach forewing showing a wrinkled, self-organized pattern at the edge. (**S**) A mite stuck to the bud, having the integument partially covered with bud secretion and distal legs entangled with secretion and trichomes. *bs* bud scale, *co* colleters, *cw* female cockroach forewing, *gl* glass, *se* secretion, *sp* self-organized pattern, *te* teflon, *tr* trichomes. Scale bars: (**A**) 200 µm; (**B**,**D**,**L**) 5 µm; (**C**,**E**,**G**,**M**,**N**,**O**,**P**) 500 nm; (**F**,**I**,**J**,**K**,**S**) 20 µm; (**H**,**Q**) 100 µm; (**R**) 2 µm.

Freeze-fractured cross-sections elucidated the amorphous nature of the secretion and a variation in thickness of the secretion layers on and between the bud scales (Fig. 3A–G). Cartwheel-shaped micelle-like inclusions of 3.7 µm in diameter were randomly found in the secretion without any specific distribution pattern (Fig. 3B,C). The filaments mentioned above could also be observed with cryo-scanning electron microscopy (cryo-SEM), which elucidated extended fibers embedded in a fluid matrix (Fig. 3D). The surface of secretion appeared rather smooth (Fig. 3E). Multicellular glands, about 70-µm wide and 50-µm high, the spherical colleters, emerged from the bud scale surface and were totally covered with the secretion (Fig. 2E–G). Removal of secretion by washing with ethanol or acetone allowed visualizing the precise position of, however, collapsed colleters (Fig. 3H–K).

### Physico-chemical properties

The secretion on the bud remained viscous throughout the whole bud growing season and even after the burst of buds under natural conditions.

**Figure 4:**
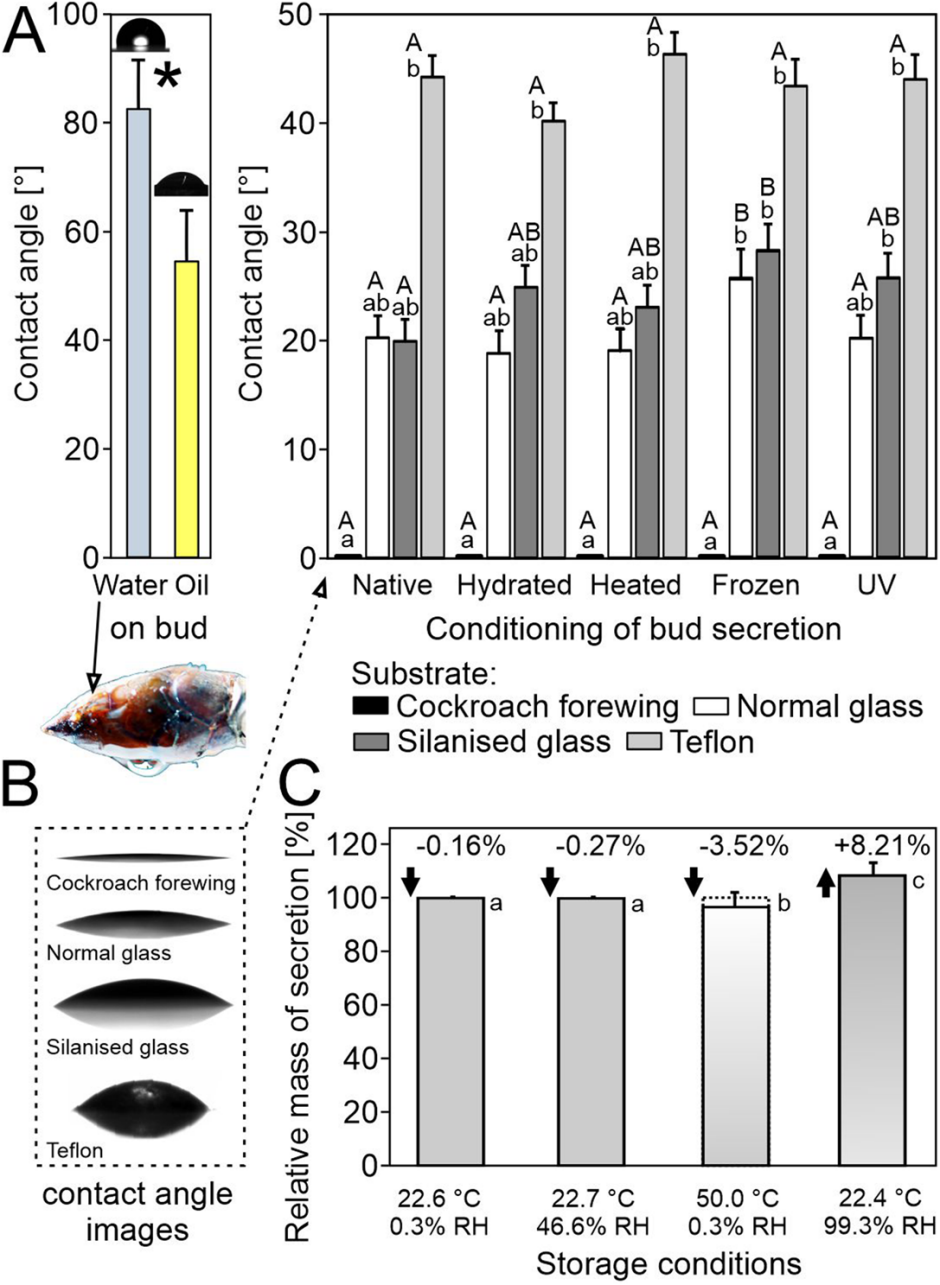
(**A**) Sessile contact angles of differently conditioned bud secretion droplets on various substrates, as well as of water and oil droplets on a native alive bud. Different small letters indicate statistical differences between substrates for the same conditioning, capital letters between conditioning for the same substrate (two-way analysis of variance, balanced design, followed by all pairwise multiple comparison procedures Tukey test, *p* = 0.05; substrate: *F*_3,199_ = 653.3, *p* < 0.001; condition: *F*_3,199_ = 4.3, *p* = 0.002; substrate and secretion conditioning did not exert interactive effects: substrate × condition: *F*_3,199_ = 1.8, *p* = 0.057). The asterisk corresponds to the statistical difference between the water and oil contact angles on bud: *t* test, *t* = − 6.3, *p* ≤ 0.001 (n = 10 per fluid). (**B**) Images of the bud secretion droplets on different substrates and a bud with an abaxially adhering droplet of water. (**C**) Relative change of the bud secretion mass on the normal glass after 7-day storage at different conditions. No significant differences in mass before and after storage for the same condition were detected (Table S3). Different small letters indicate statistic differences between treatments (Kruskal–Wallis one-way ANOVA on ranks, *H*_3,98_ = 80.1, *p* ≤ 0.001, followed by all pairwise multiple comparison procedures Tukey Test, *p* < 0.05).

The secretion was able to wet all observed substrates, establishing contact angles (CA) that in parts differed significantly from each other (Figs. 2G, 3L–S, 4A,B, Table S1). It spread over the lipoid integument of arthropods, resulting in the lowest CA of 0° (Figs. 2J–L, 3R,S, 4A,B). The effect of the substrate did not interfere with that of the secretion condition (two-way analysis of variance, balanced design, substrate × condition: *F*_3,199_ = 1.8, *p* = 0.057). After being kept in a water-saturated atmosphere, the secretion appeared slightly matt (milky) and swollen, but not dissolved (for swollen aspect see Fig. 3O). At a microscopic level, UV radiation did not cause a distinct change in secretion. Dry heating resulted in a more fluid and elastic state while freezing led to a more plastic behavior, with significantly higher CAs of secretion on glass (Fig. 4A). Single water droplets on the bud maintained a hemispherical shape (CA 82.5° ± 9.74°, mean ± sd) and did not roll-off when buds were rotated at 180° at 10 mm s^−1^ (Fig. 4B, Video S1). Oil droplets appeared significantly flatter (CA 54.6° ± 10.21°; *t* test, *t* = − 6.3, *p* ≤ 0.001). Both oil and water left distinct circular patterns on secretion after contact.

When the bud secretion was placed on glass slides and stored under different conditions for 7 days, its mass did not change significantly (Fig. 4C, Tables S2, S3). The mass decreased by less than 0.3% after storage at 22.6 °C, 0.3% RH, and 22.7 °C, 46.6% RH, and by more than 3.5% after storage at 50 °C, 0.3% RH. Under a water vapor-saturated atmosphere (22.4 °C, 99.3% RH), the secretion gained 8.2% in mass, which significantly differed from all other conditions (*p* ≤ 0.001).

**Figure 5:**
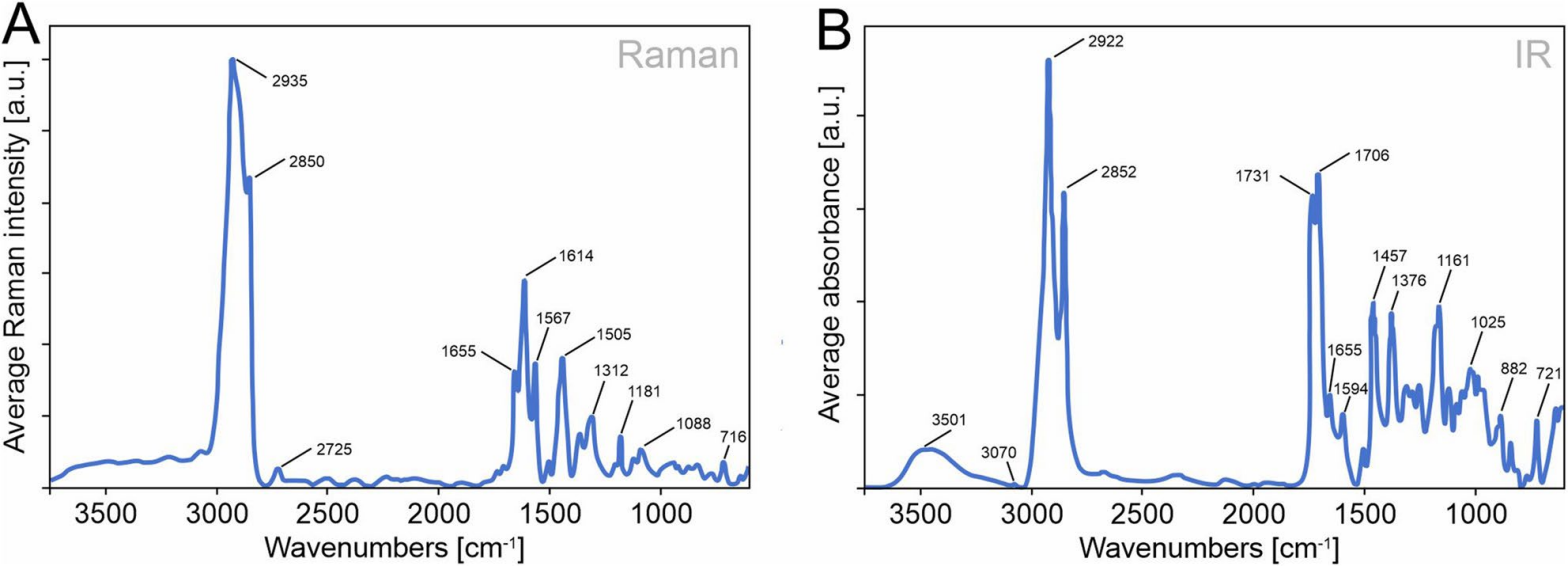
Chemical composition of the natural horse-chestnut tree bud secretion. (**A**) Raman spectrum and (**B**) ATR FTIR spectrum. Both spectra show spectral bands characteristic to OH/NH, CH_2_, C=O (ester and acid), and amide (C=O–NH) groups and thus, the presence of long-chain, saturated alkanes, fatty acids, and esters, as well as amides.

The averaged Raman (Fig. 5A) and infrared (IR) (Fig. 5B) spectra obtained for the pure non-treated horse-chestnut bud secretion represented a mixture of different biomolecules such as carbohydrates, lipids, and peptides. For this reason, bands appeared rather broad and might be a result of the spectral contribution of several different constituents. Both spectra (Fig. 5) were dominated by the presence of saturated alkanes and fatty acids and showed their characteristic band patterns (band assignment in Supplementary Table S4). Raman spectra of saturated fatty acids were characterized by typical Raman bands at 2935 and 2850 cm^−1^ (C–H asymmetric stretch assigned to CH_2_), 1443 cm^−1^ (CH_2_ bending), and 716 cm^−1^ (CH_2_ rocking)^30^. The IR spectrum of fatty acids showed bands of the alkyl chain C-H stretching and bending (2930–2800 cm^−1^; 1457 cm^−1^ and 721 cm^−1^) and the carbonyl stretch (1750–1690 cm^−1^), respectively^30^.

The C–H stretching vibration of the cis-double bond (= C–H), which are typically observed at 3005 cm^−1^ as well as at 1264 cm^−1^ (=C–H bending) and 974 cm^−1^ (out-of-plane =C–H bending), were missing, which indicates that non-saturated fatty acids or other non-saturated hydrocarbons were less likely to be present. We observed a band at 1655 cm^−1^ in both spectra, which could mean a double bond stretching vibration (C=C), typically occurring at around 1648 cm^−1^, but, most likely, to be assigned to secondary amides (R–CO–NH–R^30–32^).

The Raman and IR spectra of different fatty acids were very similar and varied only slightly with increasing chain length^33,34^. Saturated fatty acids in the solid-state have defined narrow bands, whereas the bands of short-chain fatty acids in the liquid state appear rather broad^33^. Furthermore, in Raman spectroscopy, spectral ratios are known to be useful to determine the number of C=C bonds (1655/1444 cm^−1^ ratio^33,35^) of the fatty acid chain length (e.g., the ratio between 2850/2935 cm^−1^)^34^. Taking all this into account, we suspect a rather short (< C_12_) fatty acid in the liquid state. If matched to a spectral database, the spectra showed a high resemblance to saturated fatty acids < C_10_, especially to the straight-chain saturated nonanoic (= pelargonic) acid (C_9_H_18_O_2_). This assumption was further confirmed by the Raman band intensity ratio 2850/2935 cm^−1^, which results in a value corresponding to C_9_^34^.

In addition to Raman, the IR spectrum showed two characteristic bands at 1731 and 1706 cm^−1^ in the region of the carbonyl absorption (C=O, 1750–1690 cm^−1^), indicating the presence of both esters and free carboxylic acids.

A careful examination of IR and Raman spectra revealed some significant minor components. A weak band at 3070 cm^−1^ (C–H aromatic stretch) indicated the presence of an aromatic compound, which was supported by the IR band at 1594 cm^−1^ and the Raman band at 1614 cm^−1^ that characterize C=C aromatic skeletal vibrations.

A supplementary high resolution nuclear magnetic resonance (HR NMR) spectroscopy study supported the Raman and IR findings by ^1^H and ^13^C NMR data (see Fig. S1 for method and data). Both NMR spectra were dominated by aliphatic signals (CH_2_/CH_3_) and show contributions of carboxylic acids and esters. This result is in good agreement with saturated alkanes, fatty acids, and esters being the main components. Typical chemical shifts for the N–H/H_α_ protons and the C_α_ positions in secondary amides were also observed but in minor amounts. In the NMR spectra, signals of weak intensity were found in the aromatic region, supporting the presence of a minor aromatic compound. Compared to the aliphatic signals, the other regions (carboxylic groups, non-saturated/aromatic, amides) make up less than 2% of the integral intensity of the ^1^H NMR spectrum.

### Adhesion

**Figure 6:**
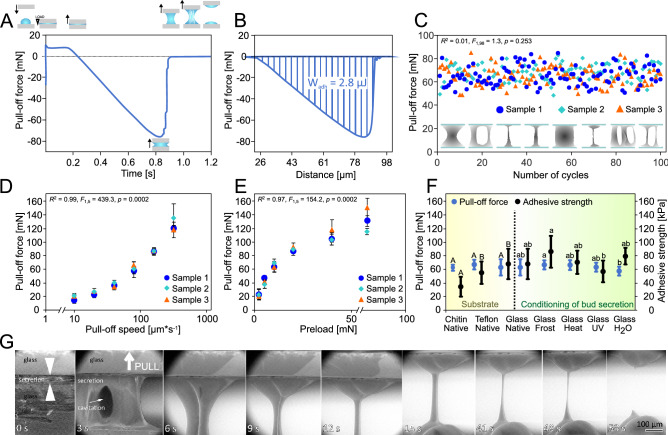
Adhesive properties of the viscous secretion released from buds of horse-chestnut trees. (**A**) An example of force–time curves obtained during pull-off force experiments with secretion placed between glass slides. The insets indicate the experimental design and measurement procedure. (**B**) A representative force-distance curve transformed from force–time data in A, displaying the work needed to separate the bud secretion between two glass slides at a distance of 50 µm. Note that also the work needed to deform the force sensor is included in this two-component measure. (**C**) Reliability of bud secretion investigated by a series of 100 repeated pull-off tests with the same sample (n = 3). Force did not decrease with the increasing number of cycles (LR, *R*^2^ = 0.01). The inset illustrates different shapes of pulled filaments of secretion observed during the experiments. (**D**) Pull-off force measured as a function of pull-off speed (means and error bars, n = 3), showing a positive relationship (NR, *R*^2^ = 0.82). (**E**) Pull-off force measured as a function of preload (means and error bars, n = 3), showing a positive relationship (NR, *R*^2^ = 0.48). (**F**) Pull-off forces and adhesive strength measured on three substrates (chitinous forewing of a female American cockroach, Teflon: polytetrafluorethylene, normal glass) and with five differently conditioned secretion samples on normal glass after keeping 5 days non-treated, frozen, heated, UV-radiated, or hydrated (means and error bars). Capitals indicate statistical differences in force or adhesive strength values between substrates; lower letters indicate statistical differences in force or adhesive strength values between secretion conditions: one-way ANOVA followed by all pairwise multiple comparison procedures Tukey test, *p* = 0.05; pull-off force @ condition: *F*_4,49_ = 2.6, *p* = 0.046; pull-off force @ substrate *F*_2,69_ = 1.1, *p* = 0.363; adhesive strength @ condition *F*_2,29_ = 3.9, *p* = 0.008, adhesive strength @ substrate *F*_2,29_ = 9.5, *p* < 0.001. (**G**) Low vacuum SEM images showing an example of pulled-off secretion within 53 s.

The pull-off force experiments proved that the horse-chestnut bud secretion is an adhesive (Fig. 6, Table S5, Video S2), which maintained its properties after 100 repeated trials (Fig. 6C), demonstrating the force ranging from 48.5 to 85 mN (64–78 kPa). The shape of obtained force–time curves indicated an abrupt loss of adhesion despite extended secretion filaments (Fig. 6A), which required a low work to be separated (Fig. 6B). The pull-off force significantly increased with increasing pull-off speed (nonlinear regression, NR, *R*^2^ = 0.99, *p* = 0.0002; Fig. 6D) and increasing preload (NR, *R*^2^ = 0.97, *p* = 0.0002; Fig. 6E), ranging between 13 and 167.5 mN. The secretion layer thickness at 10-mN preloaded condition was 9.0 ± 2.50 µm (n = 50). At the highest speed and preload (80 mN, 320 µm s^−1^), the pull-off force was 185.7 ± 17.92 mN (n = 10), corresponding to an adhesive strength of 204.3 kPa.

The pull-off force did not significantly differ between substrates (Fig. 6F). The adhesive strength was statistically two times lower on cockroach forewings compared to Teflon and glass: 34.6 ± 13.65 kPa, 55.2 ± 15.71 kPa, and 68.2 ± 21.03 kPa, respectively (one-way ANOVA followed by all pairwise multiple comparison procedures Tukey test, *F*_2,29_ = 9.5, *p* < 0.001). However, it did not depend on the secretion contact angle on a substrate (linear regression, LR, *R*^2^ = 0.07, *p* = 0.6; Fig. S2). After hydration, the pull-off force was significantly lower than that one of the frozen samples (*F*_4,49_ = 2.6, *p* = 0.046); the forces of other conditioned samples did not differ from each other. The adhesive strength was significantly lower for UV-treated samples (57.0 ± 15.35 kPa) compared to frozen ones (86.5 ± 23.23 kPa), while it did not differ between the other samples (*F*_2,29_ = 3.9, *p* = 0.008). The data set did not fit to a two-way analysis of variance and general linear model. That is why one-way ANOVA separately evaluated the groups.

Low vacuum SEM confirmed that the pull-off forces were detected at the first rupture of secretion that happened at about 20 µm displacement. Correspondingly, the fibrous nature of secretion was most prominent during the initial stages of the experiment. Further extension of filaments did not result in measurable forces and appeared as a certain flow in liquified filaments. Fast pull-off speed (> 100 µm s^−1^), lower secretion volume, heating, and hydration of samples caused extension of single long thin filaments (Fig. S4), while slower pull-off speed, freezing, and UV radiation of samples mostly implicated establishment of lamella-shaped or multifilamentous bulks of secretion (Fig. S5). Such structures frequently showed increasing cavitation with increasing displacement. After thinning, filaments separated at their middle, and the torn ends formed hook-shaped residues that snapped back to the substrate recovery droplet. Except for cockroach wings, a smaller portion of secretion adhered to the counter surface, while the larger one remained on the substrate where the sample was initially placed (Video S6). By ocular observation, frozen samples appeared to be more plastic than elastic, and they were heavier to separate. After storage at 50 °C and 0.3% RH, samples were smeary and pullable into very long thin filaments, which separated faster than those of non-treated secretion (Videos S3, S4). Hydrated secretion was more compressible and extendable into thin filaments, which snapped fast back after separation (Video S4).

## Discussion

### Structure–function relationships of the horse-chestnut bud secretion

The results observed in our study explain well why the sticky secretion impregnates and protects horse-chestnut buds over a long period while resisting a variety of environmental impacts (Fig. 1). The secretion does not dry out under arid conditions, does not melt at 50 °C, and does not change significantly under UV radiation or frost at the microscopic level. It is swellable to a certain degree underwater and in a humid atmosphere, and it universally wets substrates having different polarities and textures. Such stability over a wide range of environmental conditions is achieved by a three-dimensional polymeric network^36^, e.g., in plant resins. The latter predominantly occur in woody seed plants, being primarily lipid-soluble mixtures of volatile and nonvolatile terpenoid and/or phenolic secondary compounds^27,37^. However, the horse-chestnut bud secretion here is a multi-component material, including saturated alkanes, fatty acids, and esters, as well as polyamides, which are embedded in a fluid matrix of fatty acids/alkanes comprising nonpolar and polar portions. Low-esterified polyamides are reported to be nondegradable^38^ and to ensure high thermal stability and excellent chemical resistance to solvents, plasticizers, and oils, as well as higher mechanical flexibility on glued junctions, compared to other hot melt adhesives^39^.

Raman, IR, and NMR spectroscopy of pure bud secretion confirmed the presence of saturated alkanes, fatty acids, and fatty acid esters (most likely with a chain length < C_12_), as well as amide and aromatic constituents. Saturation explains low chemical reactivity, relatively stable consistency, and translucent appearance of bud secretion, as also previously observed by Wollenweber^24^.

Reversible changes in the secretion material properties due to UV radiation, heating, and freezing also underpin the saturated nature of secretion, which does not further polymerize or loose cross-links. At the micro and nanoscale, the bud secretion looks amorphous, well mixed, and not structured despite several micelle-shaped inclusions (Fig. 3B,C). Micelles are known to clump immiscible parts of different molecules together, increasing the miscibility and wettability of fluids^40^. Such emulsion of fluids was observed for both oil and water droplets, which left distinct circular patterns on secretion after contact. This fact refers to the biphasic composition of the bud secretion, including lipophilic (fatty) and hydrophilic (amide, ester) portions, as well as polar and nonpolar interactions in fatty acid molecules (Fig. 5A)^30,41^.

Based on the above results, saturated alkanes, free fatty acids, and fatty acid esters are the main components of the bud secretion. Fatty acids may be cross-linked and functionalized by autoxidation, polymerization, hydrogenation, hydroaminomethylation, ozonolysis, methoxycarbonylation, or polycondensation of functional monomers to polyesters^42,43^. The latter fits well with the above-mentioned nonanoic acid. Altogether, one may hypothesize an aliphatic hydrocarbon resin or oligoester to describe the complex mixture of horse-chestnut bud secretion.

In accordance with previous reports for acetone extracts of entire buds, spectroscopy in the present study provides hints for the presence of a minor aromatic compound, e.g., flavonol aglycones or rather terpenoids in the pure bud secretion^24,28,44^. Also, the recent GC–MS study with hexane extract of entire buds reporting alkanes, phenolic acid/ester, tetradecanal (C_14_H_28_O), and saturated tetradecanoic acid (C_14_H_26_O_4_)^28^ is supported to some extent by our analysis. A certain discrepancy between the results of previous and recent studies could be explained by different sampling and the inherently limited spectral resolution of Raman and IR spectroscopies due to the superimposition of spectral information from different compounds. According to Wollenweber and Jay^45^, lipophilic flavonoids co-occur with sesqui- or triterpenes in plant exudates. Terpenoids are frequently released from plant glandular trichomes, such as horse-chestnut bud colleters^27,43^, and should be included in sticky secretions resulting in instant tack, such as observed for horse-chestnut buds.

### Adhesion of the horse-chestnut bud secretion

The composition of the visco-elastic bud secretion resembles that of pressure-sensitive adhesives (PSA) consisting of the multi-component multiphase mixture of backing hydrocarbon resin and fatty acid esters, tackifying terpenes, and surfactant fatty acids, having about 50–70% of an insoluble fraction^46–48^. Bud secretion and commercial PSA share specific properties, such as a universal wetting of substrates mediating sufficient instantaneous contact formation (tack) and a four-step debon-ding process including deformation, cavitation, fibrillation, and rupture^47–50^. The bud secretion in contact spread over the substrate establishing flat layers under tension (CA of less than 30° and 50° on glass and Teflon, respectively). According to Chalykh and Shcherbina^51^, these adhesive–substrate transition zones matter in adhesive joints because they may spontaneously form emulsions of one polymer within the other polymer, creating a thermodynamic and colloid chemical interface boundary. Such a boundary was particularly observed for bud secretion on the lipoid integument of arthropods (CA of 0°), interacting with the epicuticular greasy layer, creating self-organized patterns (Fig. 3R) and a larger contact area compared to that on the other substrates (Table S5). We suggest interactive forces, which are established between the atoms and molecules at the interface of the bud secretion and substrate, fitting to the adsorption theory of material adhesion^48^.

As reported for PSA e.g.,^52^, the link formed between the substrate and the bud secretion adhesive layer further depends on the time of contact and the force applied during the bonding stage (Fig. 6D,E).

Both surface (free surface energy, roughness, etc.) and bulk properties of the adhesive are known to govern the nature and location of fracture^49,53^. Similar to technical PSAs, the natural bud secretion is a viscoelastic material that deforms under debonding force, forming cavitation bubbles and filaments of up to 5-cm long, which evince inner flow under tension (Figs. 2H,I, 3D; Videos S3–S6). Initially, cavities form on defects or trapped air at the substrate-adhesive interface when tension stress is applied. The principal role of these cavities in PSAs has been previously assigned to the reduction of local stresses and the prevention of any large stress concentrations towards the avoidance of fracture^54^. If the cavities grow laterally and vertically, they fuse and form cracks, causing rapid interfacial debonding. If the coalescence of adjacent cavities does not occur, the walls between them are extended as fibrils through the growth of interfacial crack propagation. The extension of single long thin filaments was mainly observed for high pull-off speed (> 100 µm s^−1^), low bud secretion volume, as well as heated and hydrated samples. Low pull-off speed, freezing, and UV radiation mostly resulted in lamellar or multifilamentous shapes of deformed bud secretion. After thinning, pulled filaments separated in the middle. The torn ends bent hook-shaped before they snapped back to the substrate and recovered to droplets. A typical cohesive debonding was observed, where some residues of adhesive are left on the probe at the end of the test^55^. A smaller portion of bud secretion adhered to the counter surface, while the larger one remained on the substrate where the sample was initially placed, except for lipoid cockroach wings, to which a larger residue stuck. In addition, the force–time curves obtained for bud secretion showed an abrupt decrease of adhesion despite extended secretion filaments. For PSAs, such a curve shape has been explained by the process of cavity growth in a rubbery elastic medium, which is commonly indicative of weak adhesion and interfacial crack propagation^47,54^.

As demonstrated by the consistent adhesion during 100 repeated cycles of pull-off tests, the horse-chestnut bud secretion is persistent even after cavitation, filament extension, and rupture. Such recoverable adhesive joints are mainly attributed to molecular interactions (e.g., friction between molecules^56^), rather than tight cross-links between polymer chains. The latter would avoid flow, solubility, and are commonly irreversibly broken upon rupture^47^. The recovery of bud secretion is confirmed by heat, frost, water, and UV treatments, which led to only slight changes in consistency and adhesion of secretion at the microscopic level. However, stronger intermolecular interactions are expected in frozen samples, which appear to be more plastic than elastic.

The adhesive strength was significantly higher in frozen samples compared to UV-treated ones, while it did not differ between the other samples. Thus, one may assume different effects of frost and UV radiation on bud secretion, while the latter is known for the initiation of curing by radical polymerization^55^. The storage at 50 °C and 0.3% RH loosened the bud secretion network in such a manner that samples were smeary, obviously more fluent, and pullable into very long thin filaments, which separated faster than those of non-treated secretion. Hydrated secretion appeared slightly milky, more compressible, and extendable into thin filaments, which snapped back fast after separation; the pull-off force was significantly lower than that obtained with the frost-treated samples. Such effects have been previously discussed for the sticky, resinous secretion of carnivorous dew plants *Roridula gorgonias* Planch. (Roridulaceae): the underwater changes were attributed to the polar compounds in secretion and ion interaction, causing swelling of resins and elastic materials^57–59^. Interestingly, the horse-chestnut bud secretion adhered to glass also underwater without a significant difference to maximum pull-off forces in air, reaching an average of 15.4 mN and 12.4 mN, respectively, as shown by a preliminary experiment (Fig. S3).

Commercial PSAs seem to stick more strongly than plant secretion at a comparable adhesive layer thickness. For example, probe tack tests with the standard PSA poly(2-ethyl hexyl acrylate), including 2% acrylic acid (PEHA-AA), resulted in a debonding strength of 1.3 MPa at a 1 mm s^−1^ separation rate, a 0.07–0.1 mm adhesive layer thickness, and 900 kPa applied contact pressure^60^. The maximum adhesive strength in horse-chestnut bud secretion (204 kPa) is higher than that of sticky leaf coverage in tarflowers *Befaria racemosa* Venten (Ericaceae) and commercial flypaper Tanglefoot (40–50 kPa)^61^. It resembles those obtained for pressure-sensitive adhesive secretion in dew plants *R. gorgonias* and radiator plants *Peperomia polystachya* (Ait.) Hook. (Piperaceae) on normal glass: 200 kPa and 155 kPa, respectively. Note that the applied contact pressure (kPa), layer thickness (µm), and pulling velocity (µm s^−1^) differed in previous and present experiments (*B. racemosa*: 4–9 kPa, 1300 µm, 500 µm s^−1^; *R. gorgonias*: 1.9 kPa, 130 µm, 2300 µm s^−1^; *A. hippocastanum*: 88 kPa, 9 µm, 100 µm s^−1^). Also, dew and radiator plant secretions comprise a mixture of resins based on aliphatic esters and carboxylic acids; their FTIR spectra are comparable to that of synthetic ethylene–vinyl acetate^62^. The presence of minor amounts of amide groups in concert with fatty acids in horse-chestnut bud secretion differs from other PSAs in distantly related plant species. This smart blend of multiple compounds add to the plant fitness and energy efficiency by supplying the buds of the ancient eudicot *A. hippocastanum*^63^ with a long-term persistent protective adhesive layer, having universal reliability and applicability under a wide range of impacts. How far similar effects are found in the bud secretion of other plant species remains an exciting question to be answered in future comparative studies. Also, the detailed structural chemistry and changes of bud secretion under various conditions at the molecular level need further clarification.

In the context of bio-inspired adhesion, this composition may find use in developing novel amide group-containing commercial resins to improve existing photostable polyisocyanate resins widely used for varnishes^64^, acrylate PSA used for drug delivery via hydrogen bond-forming ability^65^, functionalized biodegradable polymers^66^, and polyamide hot melt adhesives applied in the automotive, textile and electronic industries^39^.

## Conclusion

The secretion of horse-chestnut buds is a persistent, reliable, reversible, and universal PSA able to withstand numerous abiotic and biotic environmental impacts (Fig. 1). The integration of viscid and other properties, such as non-degradability, protection against water loss and high/low temperatures, is of great interest to the development of multifunctional biomimetic adhesives.

Elucidating the elemental chemical composition and adhesive properties of horse-chestnut bud secretion, our study adds to the comprehensive understanding of the diversity of adhesive plant secretions, shedding light on particular structure–function relationships. The bud secretion robustness matters for coevolutionary and ecological interactions and provides a living example for bio-inspired innovations in commercial PSA.

## Materials and methods

### Plant material

Fresh buds of *Aesculus hippocastanum* L. (Sapindaceae) were collected from about 30-year-old trees growing at ruderal sites in Dresden, Germany (51°1′30″ N, 13°43′59″ E) and kept turgescent during the study.

### Substrates

Prior to experiments, soda-lime glass microscope slides (76 × 26 mm) and 5-mm diameter circular coverslips (Gerhard Menzel B.V. & Co. KG, Braunschweig, Germany) were cleaned by immersion in Piranha solution (a mixture of sulphuric acid H_2_SO_4_ and hydrogen peroxide H_2_O_2_, 3:1), repeatedly rinsed with Aqua Millipore water and dried immediately using compressed air. Silanized object slides (Silane-Prep glass slides coated with aminoalkyl silane, Sigma-Aldrich, Saint Louis, MO, USA) and Teflon (chemical-resistant slippery PTFE Sheet, McMaster-Carr, Douglasville, GA, USA, polished, Ra ~ 40 nm) were used as supplied.

Female American cockroaches *Periplaneta americana* L. (Blattodea, Blattidae) were collected in a private property in Atlanta (GA, US; 33°45′58″ N, 84°22′19″ W) and anesthetized by freezing before cutting off their forewings using scissors. Cockroaches were selected as model arthropods because of their well-known anti-adhesive greasy lipid slough-off layer on the integument surface^67^ and large flat wings that can be utilized as sample surfaces.

### Microscopy

Secretion-covered buds were imaged with (i) a stereomicroscope Olympus SZX16 combined with objectives Olympus SDF Plapo 1.6 × PF and 0.5 × PF, the camera Olympus DP26, and the cellSens Standard software (Olympus Corp., Tokyo, Japan)^68^, (ii) the Keyence digital microscope VHX970F (Keyence Corp., Osaka, Japan), and (iii) the cryo-scanning electron microscope (cryo-SEM) SUPRA 40VP-31-79 (Carl Zeiss SMT Ltd., Oberkochen, Germany) equipped with an EMITECH K250X cryo-preparation unit (Quorum Technologies Ltd., Ashford, Kent, UK) following Bräuer et al*.*^68^. For cryo-SEM, pieces of bud scales were cut with a razor blade and mounted on metal stubs using polyvinyl alcohol (Tissue-Tek, OCT, Sakura Finetek Europe BV, Alphen aan den Rijn, the Netherlands). Subsequently, the samples were shock-frozen in liquid nitrogen in the slushing chamber, transferred to the cryo-preparation chamber at − 140 °C, sublimed for 30 min at − 70 °C, sputter-coated with platinum (layer thickness ca. 6 nm), transferred to the SEM, and then examined in a frozen state at 5 kV accelerating voltage and − 100 °C temperature. Cryo-SEM micrographs were taken using the software Smart SEM 05.03.05 (Carl Zeiss SMT Ltd., Oberkochen, Germany). Metric characteristics were determined from the obtained micrographs using the SigmaScan Pro 5.0.0 software (SPSS Inc.).

### Weighing

Secretion droplets were weighed with the Excellence balance XA 205 Dualrange (Mettler Toledo GmbH, Greifensee, Switzerland) to quantify (1) the mass of secretion used for pull-off force tests and contact angle measurements, and (2) the loss or increase of the mass of secretion after the storage under different conditions. For the latter, secretion droplets on clean glass slides were kept for 7 days under different conditions: (i) 22.7 ± 0.87 °C and 46.6 ± 3.35% RH, (ii) 22.4 ± 0.19 °C and 99.3 ± 1.72% RH, (iii) 22.6 ± 0.22 °C and 0.3 ± 0.34% RH, (iv) 50.0 ± 0.09 °C and 0.3 ± 0.02% RH. The glass slides (i–iii) were kept on 20 mm high glass platforms in Azlon plastic boxes tightly covered with lid (100 × 100 × 50 mm) or (iv) in Duroplan borosilicate glass petri dishes (100 × 30 mm Duran Group GmbH, Mainz, Germany) at room temperature without particular treatment (i), at room temperature with an Aqua-millipore-water-layer covered bottom of boxes (ii), at room temperature with a Silica-gel covered bottom of boxes (iii), and in a heating oven (Memmert GmbH & Co. KG, Schwabach, Deutschland) (iv). The temperature and relative humidity were recorded using data loggers EL-USB-2+ (Lascar Electronics Ltd, Salisbury, Wiltshire, UK).

### Contact angle measurements

The contact angle measuring device OCA25 equipped with a high-speed CCD video system and SCA20 4.5.17 software (Data-Physics Instruments GmbH, Filderstadt, Germany) was used with Aqua millipore water, oil, and differently conditioned bud secretion on various substrates according to the free sessile drop method. Normal and silanized glass, as well as Teflon, were used as control surfaces. For each substrate and condition of secretion, the contact angle of 10 secretion droplets was measured (~ 2 µg, 0.03 mm^3^). The contact angle of 10 water and oil 2-µL droplets (ExxonMobil oil AM Core 100, Exxon Mobil Corp., Spring, TX, USA) was estimated on a horse-chestnut bud and a female American cockroach forewing. In total, 165 contact angle measurements were conducted.

### Raman spectroscopy

Using a piece of a clean glass slide, about 3 mg of secretion was sloughed-off from the living buds, placed on another clean glass slides, and directly analyzed. We applied Raman spectroscopy because it is a suitable analytical tool for a limited volume of natural (non-treated) adhesive plant secretion, facilitating focus on minimum 200 × 250 nm to average 250 × 500 nm spot size of samples.

The measurements were carried out at the Papiertechnische Stiftung (PTS, Heidenau, Germany) using a WITec alpha 300M+ confocal Raman microscope equipped with 532 nm laser excitation (WITec Wissenschaftliche Instrumente und Technologie GmbH, Ulm, Germany). This system is based on a Zeiss light microscope and was used with a 50 × objective (Zeiss EC Epiplan NA 0.75; Carl Zeiss Microscopy GmbH, Jena, Germany) using 20 mW laser power and a 600 g mm^−1^ grating. Raman spectra were obtained from visibly non-contaminated bud secretion areas using 0.5–1 s exposure time and 100 accumulations. The Raman spectrum shown is an average of five different individual spots. Before averaging, the spectra were background-corrected and normalized.

### ATR FTIR

Secretion samples were prepared as described above. Attenuated total reflection Fourier-transform infrared (ATR FTIR) spectra were recorded using a Nicolet iS 5 FT-IR-spectrometer (Thermo Scientific Inc., Waltham, MA, USA) coupled to a Golden Gate Single Reflection ATR system P/N 10563 with a diamond top plate at 45° (Specac Ltd., Orpington, Kent, UK). ATR FTIR has been previously demonstrated to be a reliable method to analyze biological samples^69,70^. A spectral resolution of 1 cm^−1^ was chosen over the spectral range of 4000–600 cm^−1^. One hundred scans were accumulated for each spectrum. The spectrum shown is an average of three different individual measurements, which were ATR and baseline corrected using the OMNIC 9 Software (Thermo Scientific Inc., Waltham, MA, USA). Database search was performed using the software OMNIC Specta 2.1 (Thermo Scientific Inc.) using the Nicolet Standard Collection of Raman Spectra and Organics by Raman sample database.

### Force measurements

A custom-built tribometer^71^ that enables flat samples to self-align^72^, and that can operate inside the Quanta 250 environmental SEM (FEI, Brno, Czech Republic) was used to measure pull-off forces. In this work, all force measurements were performed outside the SEM, and the latter, in combination with the xT microscope Control software (FEI, Brno, Czech Republic), was used only to visualize different types of contact separation with differently conditioned bud secretion on normal glass and cockroach forewing. When the tribometer was operated outside the SEM, a monochrome digital camera DMK 23UP1300 (Imaging Source, Charlotte, NC, USA) mounted on a high-magnification optical lens Zoom-12 (Navitar, Rochester, NY, USA) was used to capture videos of the glass–bud secretion interface.

Using plastic pipette tips (epT.I.P.S. Standard/Bulk 0.1–20 µL, Eppendorf AG, Hamburg, Germany; inner and outer diameter of 436 µm and 901.3 µm, respectively), single droplets of bud secretion (droplet volume 0.02 ± 0.007 mm^3^, n = 20; droplet mass 1.6 ± 0.77 µg, n = 10) were sloughed off from the bud surface or pre-collected secretion bulks on glass slides and then placed on a clean normal glass slide or on an evenly fixed female American cockroach forewing. After mounting the glass slide or the wing with the secretion droplet on the tribometer sample holder, the counter glass slide was moved perpendicular to the contact plane until a specific normal load was achieved. Next, the counter glass slide was withdrawn from the contact at a constant speed. The test stopped at a complete detachment of the glass slide when the force dropped to 0 mN at about 100 µm of displacement.

Six types of experiments were carried out: (i) a series of 100 pull-off force tests, with the same sample preloaded to 10 mN for 5 s, performed at a speed of 100 µm s^−1^, to test the reliability of secretion (N = 3 samples, n = 100 tests per sample); (ii) pull-off force tests performed as a function of the pull-off speed, comparing 10, 20, 40, 80, 160, and 320 µm s^−1^ (N = 3, n = 5); (iii) pull-off force tests performed as a function of preload applied for 5 s, comparing 2, 5, 10, 20, 40, and 80 mN; (iv) pull-off force tests performed after a preload of 10 mN for 5 s at a speed of 100 µm s^−1^ on three substrates (chitinous forewing of a female American cockroach, Teflon: polytetrafluorethylene, normal glass); (v) pull-off force tests performed after a preload of 10 mN for 5 s at a speed of 100 µm s^−1^ with five differently conditioned secretion samples placed on a normal glass after keeping it for 7 days (v1) non-treated (in air at 22.3 ± 1.00 °C and 32.3 ± 4.42% RH), (v2) froozen (in air at − 15.5 ± 2.02 °C and 51.4 ± 7.03% RH), (v3) heated (in air at 50 °C and 0.3% RH in the Thermo Scientific VACUtherm vacuum oven VT6025, Thermo Electron LED GmbH, Langenselbold, Germany), (v4) UV-irradiated at 365 nm (UVP UVGL-25, Analytik Jena US, Upland, CA, USA; in air at 22.3 ± 1.00 °C and 32.3 ± 4.42% RH), and (v5) hydrated (submersed in Aqua millipore water at 22.3 ± 1.00 °C and 100 ± 0% RH) (N = 10, n = 3); (vi) pull-off force tests performed inside the SEM with a simultaneous highly-magnified imaging of the secretion-substrate interface. The SEM was operated at a low vacuum (300 Pa), at 12.5 kV, 92–96 µA, spot size 6, and a working distance of 6–12 mm. The gaseous secondary electron detector was used to visualize the non-coated samples at a rate of one image (scan) per second. Movies were created by combining the freeze frames. The secretion layer thickness under preload was measured using the obtained SEM micrographs.

After testing, the secretion samples on the glass and cockroach wing substrates were imaged with a stereomicroscope Leica 12.5 combined with the camera Leica DFC450 and the Leica Application Suite software 4.7.1 (Leica Mikrosysteme Vertrieb GmbH, Wetzlar, Germany) to estimate the contact area using the SigmaScan Pro 5.0.0 software.

Before testing a new sample, the glass substrate was cleaned by successively rinsing it with acetone and Aqua Millipore water, and then drying it with compressed air.

### Statistics

Using SigmaPlot 12.0 software (Systat Software, San Jose, CA, USA), normally and non-normally distributed data were compared with *t* test and one-way ANOVA or Mann–Whitney rank-sum test and Kruskal–Wallis one-way ANOVA on ranks, respectively. Relationships were evaluated with linear (LR) and nonlinear regressions (NR).

## Supplementary information

Supplementary Information.

Supplementary Video 1.

Supplementary Video 2.

Supplementary Video 3.

Supplementary Video 4.

Supplementary Video 5.

Supplementary Video 6.

## Data Availability

All data needed to evaluate the conclusions in the paper are present in the article and/or the Supplementary Materials. Additional data related to this paper may be requested from the authors.
