# Supplementary material for: Robust, universal, and persistent bud secretion adhesion in horse-chestnut trees
Source: Sci Rep. 2020 Nov 4;10:16925. doi: 10.1038/s41598-020-74029-5 (PMC7642395; doi:10.1038/s41598-020-74029-5)
Supplement: Supplementary file 1 — Supplementary Information. [file 41598_2020_74029_MOESM1_ESM.pdf]

Supplementary Information

to the article

Robust, universal, and persistent bud secretion adhesion in horse-chestnut trees

by Dagmar Voigt[1*], Jaekang Kim[2], Anne Jantschke[3], and Michael Varenberg[2]

[1] Institute for Botany, Faculty of Biology, Technische Universität Dresden, 01062 Dresden, Germany; dagmar.voigt@tu-dresden.de, Orcid 0000-0003-2772-8504

[2] George W. Woodruff School of Mechanical Engineering, Georgia Institute of Technology, Atlanta, GA 30332, USA; kimjk@gatech.edu, Orcid 0000-0003-3806-3149; varenberg@gatech.edu, Orcid 0000-0001-5552-0511

[3] Bioanalytical Chemistry, Technische Universität Dresden, 01062 Dresden, Germany; anne.jantschke@tu-dresden.de, Orcid 0000-0003-1257-8830

*Correspondence: Dagmar Voigt, Institute for Botany, Faculty of Biology, Technische Universität Dresden, 01062 Dresden, Germany; dagmar.voigt@tu-dresden.de

Supplementary videos

Video S1.

A droplet of 1 µL of water remains attached to the secretion-covered horse-chestnut bud after placing it on top and rotating the bud at an angle of 180°, recorded with the digital camera Nikon D90 (Nikon Corp., Japan; F/3.5, 1/30 sec, focal width 105 mm, 24 fps; 2 times accelerated).

Video S2.

The representative close-up view of the glass-bud secretion interface during pull-off force tests visualized and recorded using the High Mag Zoom and Fixed Lenses Navitar (Navitar Inc., Rochester, NY, USA) combined with the DMK 23UP1300 monochrome industrial camera (The Imaging Source Europe GmbH, Bremen, Germany); 4x accelerated. The formation of three separate filaments and the flow of secretion material, rupture and snap-back of filament, as well as recovering of secretion droplets on the substrate are visible; however, the initial cavitation cannot be detected.

Videos S3-6.

Videos recorded during pull-off force tests by merging images obtained with low vacuum SEM, using the gaseous detector at 12.5 kV acceleration voltage, 300 Pa, 94-96 µA, spot size 6, and 6-8 mm working distance.

Video S3.

Pull-off test with natural, non-treated horse-chestnut bud secretion on glass, preloaded with 10 mN and retracted at a velocity of 100 µm s^{-1}, when no distinct cavitations are visible, and a thick filament appears to become lamella-shaped during retraction. The ultrathin layer of secretion on the glass, the fluid consistency, and certain flow of the secretion are visible. After separation, the filament snaps back and the secretion droplet recovers; 4x accelerated.

Video S4.

Pull-off test with hydrated horse-chestnut bud secretion (after storing it underwater for 5 d) placed on glass, preloaded with 10 mN, retracted at a velocity of 320 µm s^{-1}, stopped before thinning and separation at a distance of 10 µm for 10 s and 50 µm for ~25 s to observe the cavitation processes and the secretion flow. The fluid consistency and certain flow of the secretion are visible. After separation, the filament snaps back and the secretion droplet recovers; 2x accelerated.

Video S5.

Pull-off test with 50 °C-treated horse-chestnut bud secretion placed on glass, preloaded with 10 mN, and retracted at a velocity of 100 µm s^{-1}, so cavitations occur first and then quickly shape into thin filaments. 0.28-0.36 shows details of the filament. The fluid consistency and certain flow of the secretion are visible. After separation, the filament snaps back, and the secretion droplet recovers; 1.75x accelerated.

Video S6.

Pull-off test with horse-chestnut bud secretion placed on cockroach wing (lower) brought in contact with a glass slide (upper), preloaded with 10 mN, and retracted at a velocity of 100 µm s^{-1}, so cavitations occur first and then followed by a thin filament. The fluid consistency and certain flow of the secretion are visible. After separation, the filament snaps back and the secretion droplet recovers. The main residue of the secretion is left on the cockroach wing and a lower volume on the glass slide, which is focused in the last 5 s in the video; 4x accelerated.

Supplementary Figures

High resolution (HR) nuclear magnetic resonance (NMR) spectroscopy of horse-chestnut bud secretion

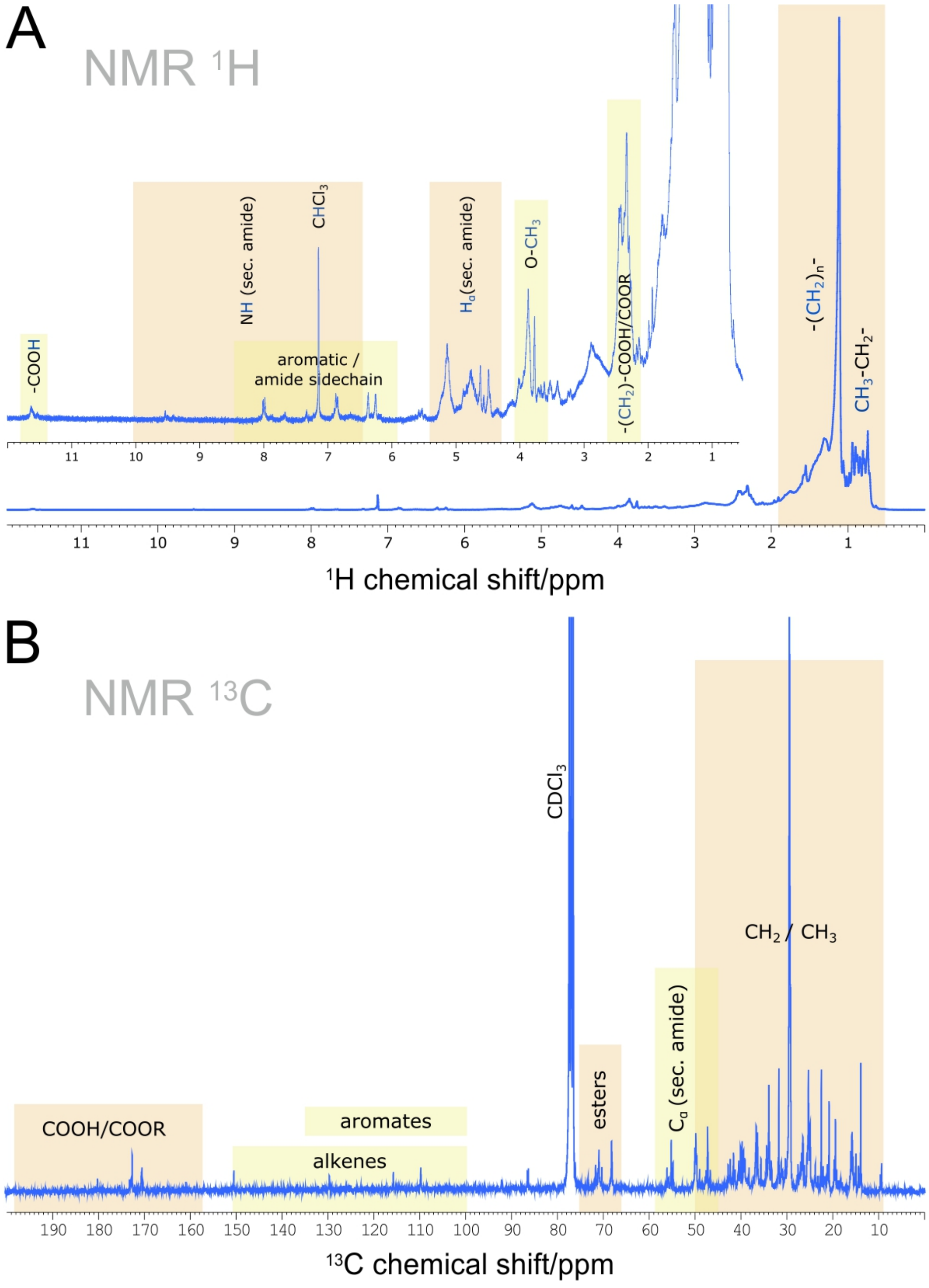

Figure S1. **A**. ^{1}H and **B**. ^{13}C HR NMR spectra (300 MHz). The ^{1}H chemical shift region >2 ppm is enlarged to demonstrate the presence of minor components (inset in A).

NMR procedure: Secretion deposited on clean glass slides was carefully dissolved in $CDCl_3$ (99.8 at-%, Aldrich). The solution had a yellow-brown color and was centrifuged

(13.000 rcf, 5 min) to remove any particles before the supernatant was transfered into the NMR test tube.
^{1}H and ^{13}C liquid-state HR NMR spectra were recorded on a Bruker Ascend 300 wide-bore NMR spectrometer (Bruker BioSpin Corp.) using a 5 mm HR probe. The resonance frequency was 300.13 MHz for ^{1}H and 75.47 MHz for ^{13}C NMR. The ^{1}H and ^{13}C chemical shifts were referenced using TMS. For ^{1}H NMR spectrum, 256 scans were accumulated with a pulse repetition time of 1 s. For ^{13}C NMR spectrum, 16384 scans were accumulated. The pulse repetition time was 5 s and ^{1}H-decoupling was applied.
Please note that in comparison to IR and Raman spectroscopy, one of the disadvantages of NMR (and mass spectroscopy) is that the adhesive cannot be analyzed in situ and must be prepared (solved). Thus, the sample might alter and/or slightly contaminated with other washed-off plant material. For this reason, the dissolved secretion probably includes constituents which are not part of the adhesive, e.g., from glands (colleters), cellular layers of bud scales, and/or bud-internal trichomes.

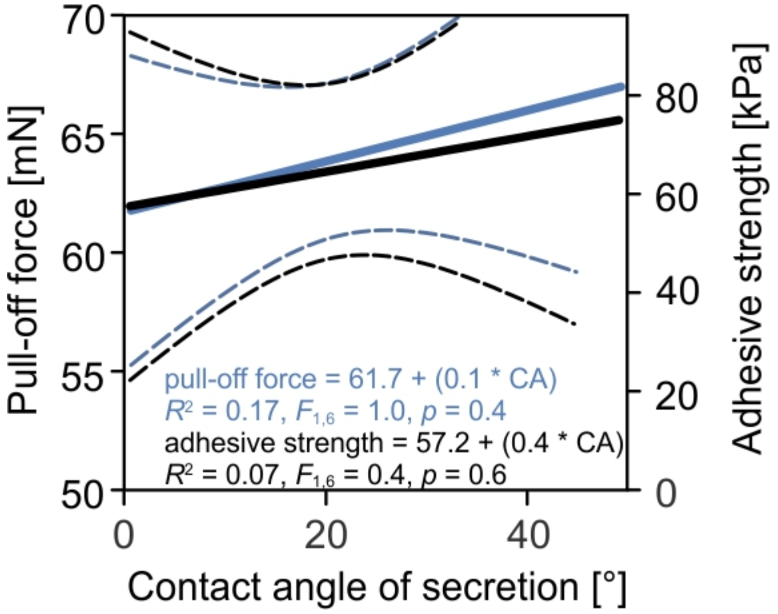

Figure S2. Pull-off force and adhesive strength versus contact angle of secretion; linear regressions (solid lines). Dashed lines specify the confidence bands at a 95% default confidence level.

In-air versus underwater adhesion of horse-chestnut bud secretion

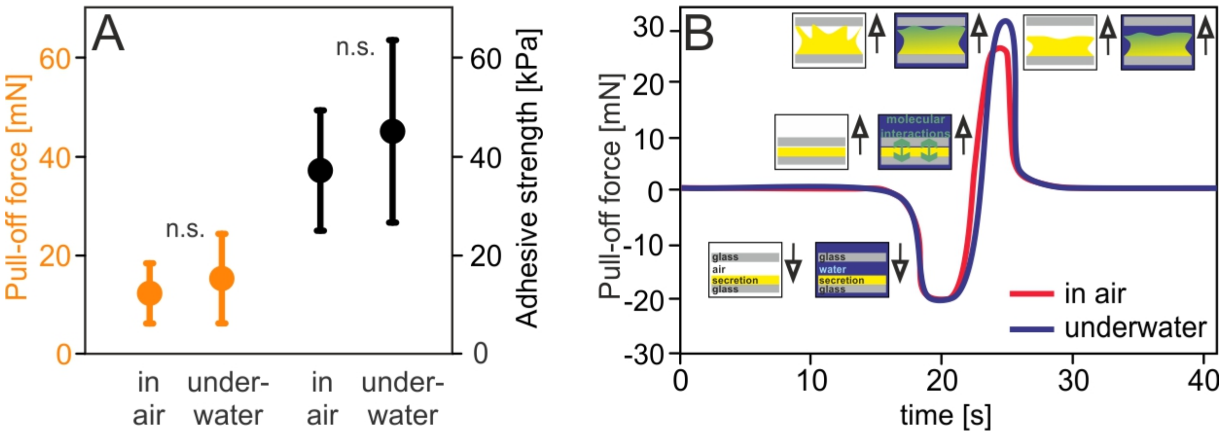

Figure S3. **A**. Pull-off force and adhesive strength of secretion on hydrophilic Piranha-cleaned glass slides (contact angle of water: 25°) in air and underwater. No significant differences between measured values in air and underwater (paired *t*-test, *df* = 9, *n* = 10; pull-off force: *p* = 0.08, *t* = 2.0; adhesive strength: *p* = 0.11, *t* = -1.8). **B**. Representative force-time curves were recorded in air and underwater. The insets illustrate the measurement procedure at the glass-secretion-air/water-interface.

Results were obtained in pull-off force tests using a FORT-25 force transducer (25 g capacity, World Precision Instruments Inc., Sarasota, USA) combined with a Lab-Trax-4/16 (LT4/16-5)-Transbridge 4M system (World Precision Instruments Inc., Sarasota, USA), according to Xu et al. 2019 (Adv. Sci. 2019, 6, 1802077). The force transducer was attached to a motorized DC3314R micromanipulator with an MS314 controller (World Precision Instruments, Sarasota, FL, USA), which was moved with the force transducer at a constant speed of 50 µm s^{-1}. The bud secretion was placed on a glass slide fixed to a holder, either kept in air or submersed in Aqua millipore water. Then, a cover slide attached to the force transducer was brought in contact with 3 µg volume of the hydrogel sample situated on the cover slide fixed on the holder at a preload of 20 mN. The thickness of the hydrogel layer was about 10 µm. Subsequently, the glass slides, and thus, the bud secretion in between them were pulled apart until the bud secretion ruptured, leaving residues on both glass slides. Using the software LabScribe2 2.248000 (iWorx Systems Inc., Dover, U.K.), force-time curves (B) were recorded to estimate the maximal traction force produced by single pull-off runs. The adhesive strength (kPa) of the bud secretion was calculated as the ratio of the maximum pull-off force to the contact area of the bud secretion on cover slide at the initial force measurements (confocal light microscopy). No statistical differences in pull-off forces and adhesive strength between air and underwater were detected. The extension of bud secretion between glass slides during the pull-off tests was ocularly observed. Interestingly, the secretion pulled into bulks and up to 15 mm thin filaments in air, while no or very tiny filaments (2-5 mm) occurred underwater. Obviously, a certain interaction (mixing/swelling) between the secretion and water took place, which is indicated by the green coloration of secretion in the insets in B. Laboratory conditions during the experiment: 22.8 ± 0.47 °C, 54.9 ± 5.49 % RH.

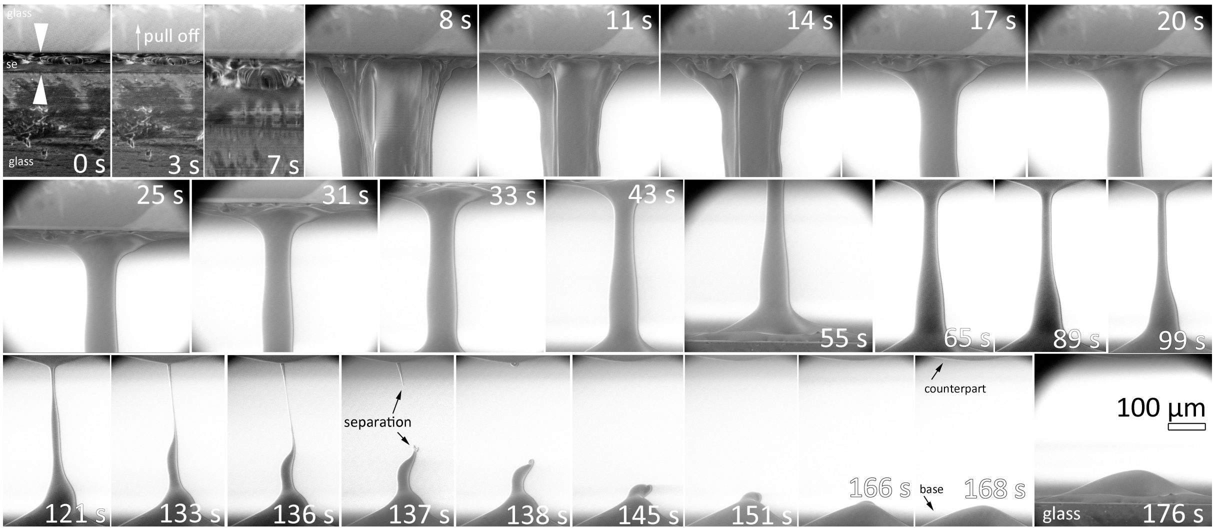

Figure S4. A series of successive low vacuum SEM images showing an example of bud secretion placed between the normal glass, preloaded at 10 mN, and separated at a speed of 320 µm s^{-1} to a distance of 50 µm. One filament is formed; it is thick and fibrous at the beginning of pull-off (8-14 s) but becomes continuously thinner during pulling until it is broken (137 s). The torn ends of filaments snap back to the secretion droplets on the substrate (138-166 s), while a more massive remnant of secretion is left on the site, where the sample was initially placed (168 s, 176 s).

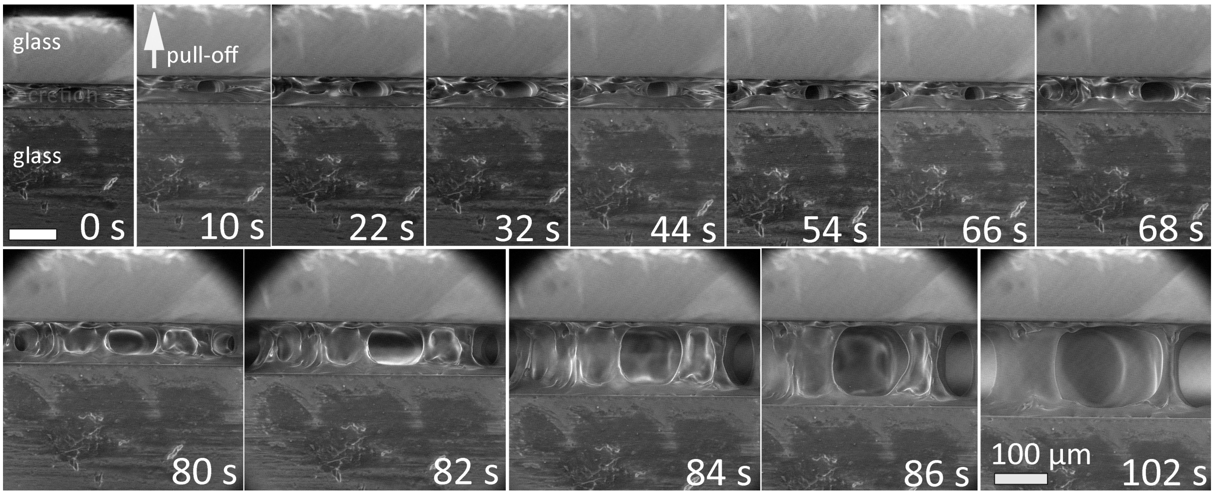

Figure S5. A series of successive low vacuum SEM images showing an example of bud secretion placed between normal glass, preloaded at 10 mN and separated at a speed of 100 µm s^{-1} to a distance of 20 µm. A bulk of secretion, including cavitations, forms during pull-off where cavitations enlarge with increased distance.

Supplementary Tables

Table S1. Contact angles of 2-μL droplets of water or oil and horse-chestnut tree bud secretion on various substrates (mean ± sd; n = 10, *n = 5) measured under laboratory conditions of 22.7 °C temperature and 46.6 % RH.

Liquid	Substrate	Contact Angle [°]
Aqua Millipore water	Normal glass	29.8 ± 4.11
	Silanized glass	63.6 ± 4.05
	Teflon	106.3 ± 4.02*
	Forewing of a female American Cockroach	74.7 ± 6.95
	Horse-chestnut tree bud	82.5 ± 9.75
ExxonMobil oil	Horse-chestnut tree bud	54.6 ± 10.21
Bud secretion not treated	Normal glass	20.3 ± 2.82
	Silanized glass	19.9 ± 6.83
	Teflon	44.3 ± 6.18
	Forewing of a female American Cockroach	0 ± 0
Bud secretion after hydration at 22.4 °C, 99.3 % RH	Normal glass	18.8 ± 2.66
	Silanized glass	24.9 ± 10.21
	Teflon	40.2 ± 9.00
	Forewing of a female American Cockroach	0 ± 0
Bud secretion after heating at 50.0 °C, 0.3 % RH	Normal glass	19.1 ± 5.43
	Silanized glass	23.1 ± 9.83
	Teflon	46.4 ± 8.76
	Forewing of a female American Cockroach	0 ± 0

Table S2. Mass of bud secretion on the glass before and after the seven-day long storage under different conditions (mean ± sd, n = 25). For statistics, see Table S2.

Condition (Treatment)	Mass before treatment [mg]	Mass after treatment [mg]	Portion of initial mass [%]
22.7 °C, 46.6 % RH	11.2 ± 0.00	8.4 ± 0.01	99.7 ± 0.54
22.4 °C, 99.3 % RH	3.8 ± 1.34	4.1 ± 1.45	108.2 ± 4.46
22.6 °C, 0.3 % RH	3.7 ± 1.40	3.7 ± 1.37	99.8 ± 0.52
50.0 °C, 0.3 % RH	5.4 ± 1.65	5.2 ± 1.71	96.5 ± 5.34

Table S3. Statistical parameters for horse-chestnut tree secretion droplet mass before vs. after seven-day long conditioning on the normal glass. Mann-Whitney rank-sum test; T, T statistics, p, probability value. For mean values, see Table S1.

Condition	T	p
22.7 °C, 46.6 % RH	711.5	0.106
22.4 °C, 99.3 % RH	584.0	0.304
22.6 °C, 0.3 % RH	641.0	0.954
50.0 °C, 0.3 % RH	674.0	0.485

Table S4. IR and Raman band assignment; major compounds in bold letters.

Wavenumber [cm^{-1}]		Band assignment	IR intensity	Raman intensity
3600−3200		ν_{O-H} or ν_{N-H}	w	
3070		ν_{C-H} (aromatic)	vw	vw
**2922**		**ν_{as} (CH_2)**	s	
	**2929**			s
**2852**		**ν_{s} (CH_2)**	m	
	**2854**			s
	2725	ν_{C-H} (aldehyde)		w
**1731**		**$\nu_{C=O}$ (ester)**	m-s	
**1706**		**$\nu_{C=O}$ (free carboxylic acid)**	m-s	
**1655**		**$\nu_{C=O}$ (secondary amide)** or **ν**$_{C=C}$ (fatty acid)	w	w-m
	**1614**	**ν**$_{C=C}$ (aromatic)		m
1594			w	
	1567	COO-		w-m
1501			w	
1457		δ (CH_2)	w-m	
	1443			w-m
1376		δ (CH_3)		
	1367			w
	1312	C-H twisting		w
1253		**ν**$_{C-O-C}$ (aromatic)		
**1176**	1181	**ν_{as}** (C-O-C) **(fatty acid esters)**		
**1161**				
	1088	**ν_{s}** (C-O-C) **(fatty acid esters)**		
1082				
882		region 822–866 cm^{1} assigned to the CAH, CAOAH, and CH2 bending		
839				
**721**		δ_{C-H} (CH_2 rocking/*oop* aromatic)		
	716			

vw, very weak; w, weak; m, medium; st, strong; vst, very strong

Table S5. Pull-off force, adhesive strength, and contact area of the bud secretion droplets measured on different substrates with differently conditioned bud secretion (mean ± sd, N = 10, n = 3), measured under laboratory conditions of 22.7 °C temperature and 46.6 % RH.

Substrate	Conditioning of secretion	Pull-off force [mN]	Adhesive strength [kPa]	Contact area of secretion [µm^2]
Normal glass	22.7 °C, 46.6 % RH	63.0 ± 11.08	68.2 ± 21.03	979.1 ± 231.39
	22.4 °C, 99.3 % RH	57.6 ± 6.39	79.6 ± 11.89	740.5 ± 151.99
	50.0 °C, 0.3 % RH	66.5 ± 8.42	70.7 ± 17.02	990.5 ± 248.42
	-15.5 °C, 51.4 % RH	66.8 ± 7.83	86.5 ± 23.23	821.6 ± 215.74
	22.7 °C, 46.6 % RH + UV radiation	63.4 ± 7.23	57.0 ± 15.35	1186.1 ± 324.72
Teflon	22.7 °C, 46.6 % RH	67.3 ± 7.25	55.2 ± 15.71	1281.8 ± 258.71
Forewing of a female American cockroach	22.7 °C, 46.6 % RH	62.9 ± 5.10	34.6 ± 13.65	2121.0 ± 864.31
